# Evaluation of the use of a heparin dose‐response test in dogs to determine the optimal heparin dose during intravascular procedures and assessment of the in vitro heparin response in healthy dogs

**DOI:** 10.1002/vms3.1326

**Published:** 2023-11-21

**Authors:** A. Hellemans, N. Devriendt, L. Duchateau, K. M. J. Devreese, F. De Somer, T. Bosmans, G. Mampaey, P. Smets

**Affiliations:** ^1^ Faculty of Veterinary Medicine Small Animal Department Ghent University Merelbeke Belgium; ^2^ Faculty of Veterinary Medicine Biometrics Research Center Ghent University Merelbeke Belgium; ^3^ Coagulation Laboratory Faculty of Medicine Department of Diagnostic Sciences Ghent University Hospital Ghent University Ghent Belgium; ^4^ Faculty of Medical Sciences Department of Cardiac Surgery Ghent University Ghent Belgium; ^5^ Experimental Research Laboratory of Cardiac Surgery and Circulatory Physiology Faculty of Medical Sciences Ghent University Ghent Belgium

**Keywords:** activated clotting time, anti‐Xa, canine, heparin resistance, heparin sensitivity

## Abstract

**Background:**

No guidelines for administering and monitoring anticoagulants intraprocedurally are currently available in dogs, despite the prevalence of procedures necessitating systemic anticoagulation with heparin.

**Objectives:**

To evaluate an activated clotting time (ACT)‐based heparin dose‐response (HDR) test to predict the individual required heparin dose in dogs during intravascular procedures, and to investigate both the in vitro heparin – ACT and in vitro heparin – factor anti‐Xa activity (anti‐Xa) relationships in dogs.

**Methods:**

Blood was collected from eight healthy beagles undergoing a cardiac procedure and utilised to establish baseline ACT and for in vitro evaluation. Subsequently, 100 IU/kg heparin was administered intravenously (IV) and ACT was remeasured (HDR test). The required heparin dose for an ACT target response ≥300 s was calculated for each individual and ACT was remeasured after administration of this dose. For in vitro testing, a serial heparin blood dilution (0‐0.5‐1‐2‐4 international unit (IU)/mL) was prepared and ACT and anti‐Xa were determined using whole blood and frozen plasma, respectively.

**Results:**

The HDR test overestimated the required heparin dose in 3/7 dogs. In vitro, ACT and anti‐Xa increased significantly with increasing blood heparin concentration. Heparin – ACT was nonlinear in 4/8 dogs at heparin concentrations >2 IU/mL, whereas heparin – anti‐Xa remained linear throughout the tested range.

**Conclusions:**

The HDR test poorly estimated the required heparin dose in dogs. This is most likely attributed to a nonlinear heparin – ACT relationship, as observed in vitro. Anti‐Xa is a promising alternative for ACT; however, unavailability as a point‐of‐care test and lack of in vivo target values restrict its current use.

## Graphical abstract

This study evaluated an activated clotting time (ACT)‐based heparin dose‐response (HDR) test to predict the required heparin dose in dogs and investigated the in vitro heparin – ACT and in vitro heparin – factor anti‐Xa activity (anti‐Xa) relationships in dogs. The HDR test poorly estimated the required heparin dose, most likely due to a nonlinear heparin – ACT relationship, as observed in vitro. Anti‐Xa is a promising alternative for ACT; however, unavailability as a point‐of‐care test and lack of in vivo target values restrict its use.

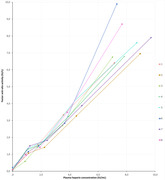

## 1 INTRODUCTION

Inadequate anticoagulation during intravascular procedures increases the risk for thromboembolic events and postoperative bleeding in humans (Shore‐Lesserson et al., 2018; Sticherling et al., 2015). Unfractionated heparin is the most commonly administered systemic anticoagulant during intravascular procedures in humans and dogs (Finley & Greenberg, 2013; Pelosi et al., 2013). The point‐of‐care (POC) test, activated clotting time (ACT), has been extensively used in human patients to monitor the intraoperative anticoagulant effects of heparin (Finley & Greenberg, 2013). The growing number of intravascular procedures currently performed in dogs, including cardiopulmonary bypass (CPB), creates a similar need for heparin monitoring in this species (Pelosi et al., 2013). Targeted heparin dosages during these procedures may often generate coagulation times exceeding the detection limit of other available haemostatic tests, such as activated partial thromboplastin time (aPTT). Yet, they remain within the measuring range of ACT tests (Hyatt & Brainard, 2016).

The ACT test is performed on venous or arterial noncoagulated whole blood and is based on stimulation of the intrinsic clotting cascade by a contact activator such as diatomaceous earth or kaolin (Finley & Greenberg, 2013; Shore‐Lesserson, 2008). The test result is expressed as the time in seconds (s) between contact activation and measurable clot formation. Depending on the specific procedure and clinical context, various recommended safe target levels for ACT have been suggested for use in humans. Owing to the substantial variation in heparin sensitivity among human patients, achieving the intended target may require a broad range of heparin doses (Finley & Greenberg, 2013). Consequently, ACT‐based heparin dose‐response (HDR) testing is often performed in humans to determine the patient's sensitivity to heparin by assessing the response to a fixed dose (Bull et al., 1975; Shore‐Lesserson, 2008). This individual heparin sensitivity is then used to compute the required heparin dose for a given ACT target value. Some contemporary ACT analysers such as Hepcon HMS plus or Hemochron RxDx offer automatic HDR testing as a feature (Garvin et al., 2010). Crucial to the validity of HDR tests, is a linear heparin − ACT relationship within the desired range. In humans, heparin – ACT linearity has been described in vivo (Bull et al., 1975; Despotis et al., 1996). However, it has been suggested by some authors that the duration and intensity of the anticoagulant response measured by ACT may not be linear at high doses (i.e. >300 IU/kg) (de Swart et al., 1982; Finley & Greenberg, 2013; Garvin et al., 2010; Hirsh, 1991; Levy et al., 2000). In spite of the latter, ACT remains by far the most commonly used coagulation test in humans to monitor heparin response during intravascular procedures (Finley & Greenberg, 2013; Pelosi et al., 2013; Shore‐Lesserson, 2008).

To date, most of the available literature on heparin administration and monitoring during intravascular procedures in dogs is based on individual case reports/series, using fixed‐dose protocols. Some of these studies failed to include any form of therapeutic effect monitoring such as ACT. In addition, scientific evidence behind anticoagulatory targets is lacking and practices in veterinary species have been adopted from human medicine (Pelosi et al., 2013). Reported target ACT in dogs undergoing CPB show significant variation, with values ranging from over 240 to over 800 s (Pelosi et al., 2013). As a general guideline, higher targets are usually preferred for cardiac procedures associated with a higher risk for thromboembolic events, such as CPB. However, clinicians should take into account other factors, including the anticipated duration of the procedure, the anatomical location (left or right side of the heart), and other prothrombotic risk factors associated with the patient (Doganer et al., 2021; Sticherling et al., 2015).

More recently, some authors have investigated the feasibility of using factor anti‐Xa activity (anti‐Xa) tests to monitor heparin therapy in humans during cardiac surgery (Reyher et al., 2016). Anti‐Xa is determined using a laboratory chromogenic assay and the test result is expressed in international units (IU) of anti‐Xa activity per mL (Reyher et al., 2016; Rosenberg et al., 2010). Anti‐Xa is considered the ‘gold standard’ for measuring plasma heparin concentrations in humans and is much less susceptible to nonheparin influences than aPTT (Gehrie & Laposata, 2012; Vermeiren et al., 2022). It is therefore currently preferred over traditional aPTT test for monitoring nonprocedural heparin therapy (Devreese et al., 2015; Garcia et al., 2012; Gehrie & Laposata, 2012; Levy & Connors, 2021; Reyher et al., 2016; Rosenberg et al., 2010; Vermeiren et al., 2022). The reasoning behind the expanded use of anti‐Xa for intravascular procedures such as cardiac surgery stems from the fact that anti‐Xa measurements have been found to correlate better with circulating heparin concentrations during CPB than ACT in humans (Reyher et al., 2016). However, there are still important practical limitations to the use of anti‐Xa measurement that favour ACT for this purpose, such as its unavailability as a POC test and the lack of safe procedural targets. The effectiveness of anti‐Xa for long‐term monitoring of subcutaneous heparin administration in dogs has been reported, but its potential for guiding intravenous (IV) heparin therapy during intravascular procedures remains unexplored (Helmond et al., 2010).

The first aim of this canine study was to evaluate the in vivo utility of an ACT‐based HDR test in predicting the required heparin dose in individual dogs during cardiac procedures. The second aim was to investigate the in vitro relationship between whole blood heparin concentrations and ACT over a range of clinically relevant heparin concentrations. The third aim was to investigate the in vitro relationship between plasma heparin and anti‐Xa over the same range of heparin concentrations.

## 2 MATERIALS AND METHODS

### 2.1 Animals

Eight healthy research Beagle dogs requiring intraprocedural heparinisation for an experimental intravascular cardiac procedure (validation of an electrophysiology protocol) were included. Dogs were considered healthy after undergoing physical examination, serum biochemistry and complete blood count. The study protocol was approved by the local Ethical Committee (EC 2021–031).

### 2.2 In vivo study protocol

Dogs received methadone premedication (0.1 mg/kg IV), followed by coinduction with midazolam (0.3 mg/kg IV) and propofol (2–8 mg/kg to effect IV). Anaesthesia was maintained using isoflurane vaporised in 100% oxygen with a circle rebreathing system. Following this, an 8 or 9 Fr. jugular vein sheath was placed. A total of 5.2 mL of blood was withdrawn. Five millilitres were placed in a plastic test tube without additives for immediate in vitro testing (protocol displayed below). The remaining sample was utilised to determine the baseline ACT (ACT_0_) using the i‐STAT 1 (i‐STAT 1, Abbott Point‐of‐Care, Belgium) and compatible kaolin cartridges (ACTk test cartridge, Abbott Point‐of‐Care, Belgium). The i‐STAT 1 has a reported measurement range between 50 and 1000 s. Earlier use of the i‐STAT 1 in dogs suggest good analyser reliability (intraclass correlation coefficient of 0.87 and coefficient of variation of 3.3%) and low intrasubject within‐day variability (coefficient of variation 8.1%) for ACT (Hellemans et al., 2023). Subsequently, a single dose of 100 IU/kg unfractionated heparin (Heparin 5000 IU/mL, Leo Pharma, Belgium) was administered IV. Ten minutes later, ACT was remeasured (ACT_100_). Based on both ACT measurements, the individual heparin sensitivity (s per IU) was calculated using the following formula described by Finley and Greenberg (2013):

$$\mathrm{Sensitivity}\;=\;\frac{{\mathrm{ACT}}_{100}-{\mathrm{ACT}}_{0}}{\begin{array}{c} \mathrm{Body}\;\mathrm{weight}\;\times \;100\frac{\mathrm{IU}}{\mathrm{kg}} \end{array}}.$$

Subsequently, the required heparin dose (IU) was calculated for a target ACT response of 300 s. This target was selected in adherence with the recommendations set by the European Heart Rhythm Association guidelines regarding antithrombotic management during electrophysiological procedures (Sticherling et al., 2015). The following formula was used to calculate the individual required heparin dose for this target (Finley & Greenberg, 2013):

$$\mathrm{Required}\;\mathrm{heparin}\;\mathrm{dose}\;=\;\frac{300\;s\;-{\mathrm{ACT}}_{100}}{\begin{array}{c} \mathrm{Sensitivity} \end{array}}.$$

Ten minutes after administration of this individual required heparin dose, ACT was remeasured (ACT_ind_) (Sticherling et al., 2015). If the measurement was <300 s, an additional heparin bolus was administered as necessary until the target ACT ≥300 s was achieved. Activated clotting times were remeasured 10 min after administration of each additional bolus and every 30 min throughout the procedure once ACT was ≥300 s. Further ACT measurements and heparin administrations were excluded from the current analysis, as sheaths and catheters were continuously flushed with heparinised saline (10 IU heparin/mL NaCl) using pressurised bags as recommended for electrophysiology procedures in humans (Sticherling et al., 2015). Therefore, individual heparin doses could not be reliably tracked thereafter.

The protocol required amendment after administering the calculated heparin dose of 478 IU/kg to Dog 2, which led to the prolongation of ACT_ind_ >1000 s. Given this ACT response outside the measuring range of the analyser and given the uncertainty of a linear dose response in dogs, the total dose of heparin per administration was subsequently limited to 250 IU/kg. This decision was made according to the suggested loading dose for comparable electrophysiology processes in humans (90–200 IU/kg) (Sticherling et al., 2015).

If the ACT was >150 s at the end of the procedure, 10 mg of protamine sulphate (Protamine sulphate Leo, Leo Pharma, Belgium) was given IV over 10 min for every 1400 IU of heparin that was administered in the form of boluses during the procedure. No specific deduction was made for any biological clearance of heparin during the procedure so as to compensate for any additional heparin that might have been administered via continuous flushing of the sheaths. After giving the complete protamine dose, ACT was remeasured (ACT_post‐prota_), and the introducer was removed when ACT was <150 s (Kern, 2013). All dogs were admitted for 24 h for follow‐up of clinical parameters.

### 2.3 In vitro study protocol

To study the in vitro relationships between heparin and ACT in blood and heparin and anti‐Xa in plasma, a series of heparin blood dilutions were immediately prepared upon blood sampling for each dog. Heparin in 0.9% saline stock dilution (100 μL, Heparin Leo 100 IU/mL, Leo Pharma, Belgium) was used to spike 900 μL fresh nonanticoagulated blood. After carefully mixing the blood with the heparin stock dilution, the following blood heparin concentrations were obtained: 4.0, 2.0, 1.0 and 0.5 IU/mL heparin. These in vitro concentrations are the approximate in vivo heparin equivalent of 292, 146, 73 and 37 IU/kg, respectively (Levy et al., 2000). A blank blood sample diluted by 100 μL 0.9% saline (0 IU/mL heparin) was added. Subsequently, 40 μL of each dilution was used for ACT measurement with the i‐STAT 1. The remaining sample volume (960 μL) from each dilution was immediately transferred into a citrate tube (3.2% sodium citrate) and centrifuged (3500 rounds per min) for 5 min at 2°C. A minimum of 400 μL citrated plasma from each heparin concentration was stored at −80°C for anti‐Xa analysis in batch by a chromogenic assay (STA®‐Liquid Anti‐Xa assay, Stago, Asnières‐sur‐Seine, France). A reagent not containing dextran sulphate or exogenous antithrombin (AT) was used. An antithrombin activity analysis was performed (AT, BIOPHEN AT anti‐(h)‐Xa LRT Hyphen Biomed, France) in all dogs on the 0 IU/mL heparin sample.

### 2.4 Statistical analyses

The in vivo study was limited to a descriptive analysis. Data are reported as median (range) and individually tabulated (Table 1). All recorded ACT measurements >1000 s (above measurement range) were treated as 1000 s. All in vitro study analyses were performed using R version 4.1.1 (Copyright © 2021 The R Foundation for Statistical Computing). As ACT was not normally distributed, a nonparametric mixed model with dog as random effect was used to study the relationship between heparin concentration and ACT using the nparLD library. A linear mixed effect model with dog as random effect was used to study the relationship between plasma heparin concentration and anti‐Xa. The Spearman's rank correlation coefficient was calculated for each dog separately and overall for both relationships. Results were considered statistically significant if the *p* value was <0.05.

**TABLE 1:** Summary of the individual in vivo data.

Dog	ACT_0_ (s)	ACT_100_ (s)	ACT_ind_ (s)	ACT _post‐prota_ (s)	Sensitivity index (s/IU)	Calculated required heparin dose (IU/kg)	Individual administered heparin dose (IU/kg)
2	109	142	>1000	92	0.0303	478	478
3	98	153	219	103	0.0390	267	250
4	98	142	>1000	87	0.0306	360	250
5	103	142	>1000	97	0.0339	405	250
6	98	109	197	114	0.0096	1736	250
7	86	153	175	92	0.0536	219	219
8	98	125	169	98	0.0245	648	250

Abbreviations: ACT_0_, baseline activated clotting time measurement; ACT_100_, ACT measurement 10 min after administration of 100 IU/kg heparin; ACT_ind_, ACT measurement 10 min after administration of the individual administered heparin dose; ACT_post‐prota_, ACT measurement 10 min after protamine administration; IU, international unit; s, seconds.

## 3 RESULTS

### 3.1 Animals

The dogs' median age was 6.2 years (range of 4.1–7.5) with a median body weight of 12.2 kg (range of 11.2–15.7). There were six female neutered dogs and two male neutered dogs. Appendix I provides a summary of several key haematological and biochemical parameters for each dog.

### 3.2 In vivo HDR testing

Dog 1 was excluded from the in vivo study because the HDR test protocol was not completed due to failure to administer the calculated required heparin dose. The authors intended to conduct the HDR test after acquiring secure left heart access through transseptal puncture, a widely used practice in Europe for human patients (Chen et al., 2014). However, the overall duration for the HDR test (comprising of ACT_0_ and ACT_100_ measurement, along with a 10 min waiting interval following each heparin administration) surpassed the timeframe within which higher ACT levels of ≥300 s were essential for our experimental intravascular cardiac procedure. Therefore, in the ensuing seven dogs, the HDR examination was executed at the start of the procedure in order to reach the target value before attempting left heart access.

Table 1 presents an overview of the in vivo ACT measurements, heparin sensitivities, and heparin administrations for each individual dog. The median ACT_0_ for dogs was 98 s (range of 86–109). Following the first heparin dose, the median ACT_100_ was 142 s (range of 109–153). The resulting median heparin sensitivity and median required heparin dose for a target response of ≥300 s was 0.0306 s per IU heparin (range of 0.0096–0.0536) and 405 IU/kg (range of 219–1736), respectively. Upon the administration of the first 100 IU/kg of heparin (HDR test), all dogs received supplementary heparin. Two of these dogs received the required heparin dose as calculated. In the case of Dog 2, the calculated required heparin dose was 478 IU/kg which prolonged ACT_ind_ >1000 s (beyond measuring range), requiring us to change our protocol (see material and methods). In this dog, no further heparin was administered throughout the remainder of the procedure. In Dog 7, the calculated required heparin dose was 219 IU/kg resulting in an ACT_ind_ of 175 s. As the measurement was <300 s, one additional bolus (250 IU/kg) was needed. In the remaining five dogs (Dogs 3, 4, 5, 6 and 8), the calculated required heparin dose was above the 250 IU/kg upper limit and therefore, only 250 IU/kg was administered. However, even with this lower heparin dose, the resulting ACT_ind_ was >1000 s in two dogs (Dogs 4 and 5). In another three dogs (Dogs 3, 6 and 8), the resulting prolongations in ACT_ind_ were below the target of ≥300 s and one (Dog 3: 147 IU/kg; Dog 6: 150 IU/kg) or two additional subsequent boli (Dog 8: 250 + 250 IU/kg) were required until a prolongation ≥300 s was achieved.

In all dogs, heparin reversal with protamine was deemed necessary. The resulting median ACT_post‐prota_ was 97 s (range 87–114). One of the dogs, Dog 7, developed a self‐limiting hematoma at the introducer site. No other adverse effects that could be associated with the administration of heparin or protamine were observed.

### 3.3 In vitro heparin – ACT and heparin – anti‐Xa relationship

**FIGURE 1:**
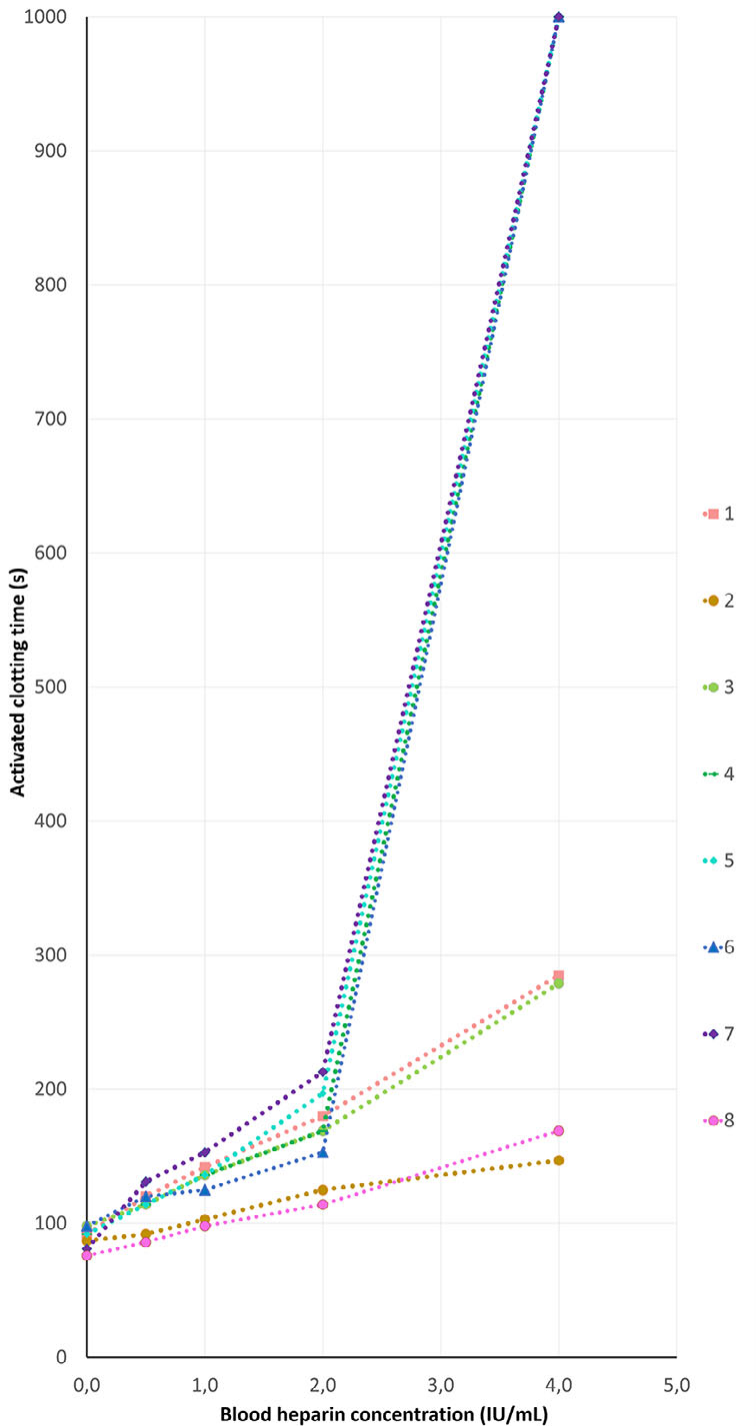
Individual in vitro relationship between blood heparin concentration and activated clotting time in eight dogs. Measurements from each dog are represented by a different colour.

Activated clotting times increased significantly with increased heparin concentration (*p* < 0.001). Spearman's rank correlations for each individual sample were all equal to 1, meaning that a higher heparin concentration always corresponded with a higher ACT value within a dog. The relationship between heparin and ACT was nonlinear in 4/8 dogs at heparin concentrations >2 IU/mL (Figure 1). The median ACT of the blank blood sample (measurement after 1/10 0.9% saline sample dilution) was 92 s (range 76–98). The variability between dogs increased with increasing heparin concentrations with the highest variability at the concentration of 4 IU/mL heparin; corresponding ACT measurements ranged from 147 to >1000 s (Figure 1).

**FIGURE 2:**
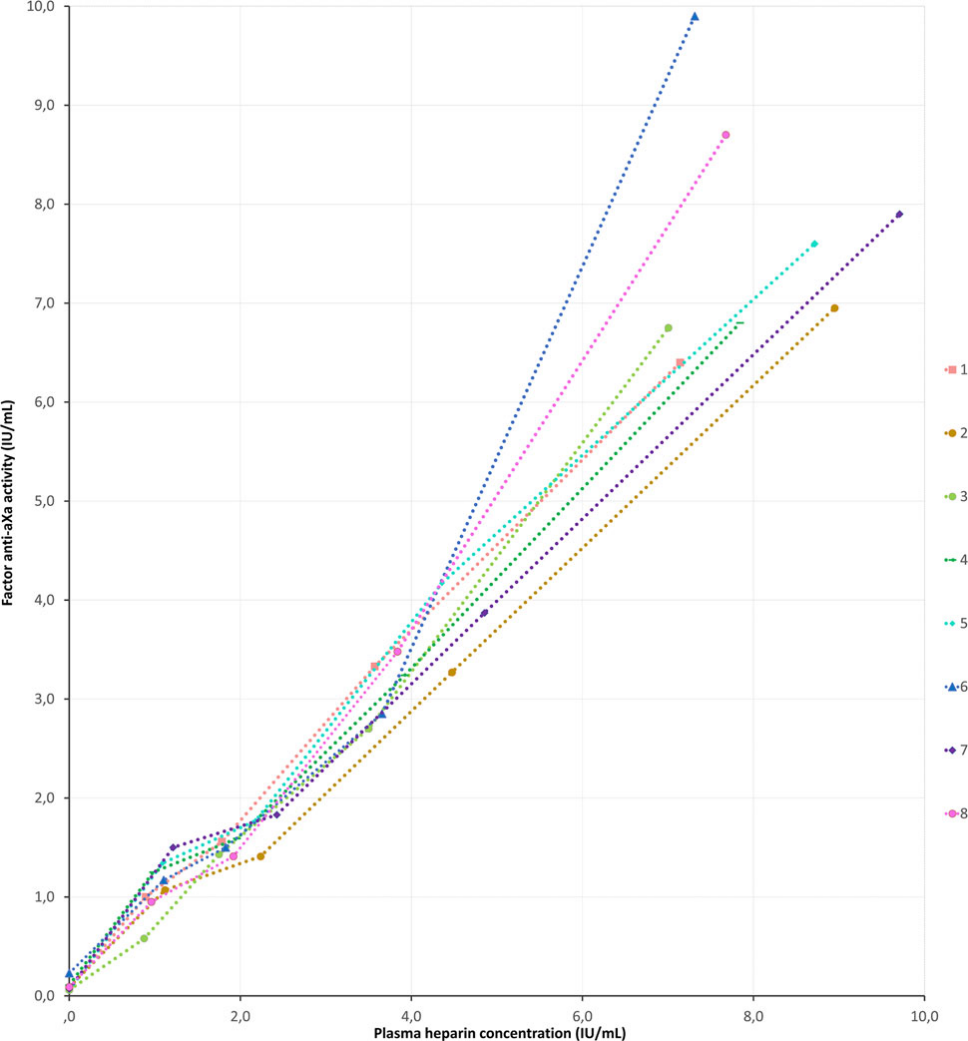
Individual in vitro relationship between plasma heparin concentration and factor anti‐Xa activity in eight dogs. Measurements from each dog are represented by a different colour.

Anti‐Xa increased significantly with increasing heparin concentrations (*p* = 0.038). Spearman's rank correlations for each individual sample were all equal to 1, meaning that a higher heparin concentration always corresponded with a higher anti‐Xa value within a dog. The relationship between heparin and anti‐Xa was linear throughout the range of tested heparin concentrations (Figure 2). No marked increase in variability between dogs was observed at high heparin concentrations as observed for ACT (Figure 2).

The median antithrombin activity of the blank blood sample (measurement after 1/10 0.9% saline sample dilution) was 107% (range of 93–112).

## 4 DISCUSSION

This study was performed to investigate the usefulness of an ACT‐based HDR test to estimate the required heparin dose in dogs during intravascular procedures and to investigate the in vitro relationship in dogs between heparin and two coagulation tests used for heparin monitoring in human medicine, namely, ACT and anti‐Xa. Our data revealed that the HDR test could not reliably predict the required heparin dose in vivo. The in vitro data showed that ACT increased significantly, but nonlinearly, with increasing heparin in blood in half of the dogs. Anti‐Xa increased significantly and linearly with increasing heparin in plasma in all dogs.

The proposed HDR test did not predict the actual required heparin dose with reasonable accuracy and overestimated the required heparin dose in at least three cases, resulting in excessive heparin administration. In two of these dogs, a heparin bolus smaller than the calculated required heparin bolus (fixed bolus of 250 IU/kg) still resulted in ACT >1000 s. Although i‐STAT ACT values >1000 s are deemed safe in humans and signify sufficient anticoagulation during CPB, such values ought to be avoided, if possible, because they denote excessive heparin dosing that could cause adverse postoperative effects such as haemorrhage and heparin rebound (Dirkmann et al., 2019; Gravlee et al., 1992; Shuhaibar et al., 2004). Heparin rebound is defined as a late postoperative increase in blood heparin concentration after apparently complete neutralisation by protamine due to the slow release of excess protein and fat‐bound heparin (Shore‐Lesserson et al., 2018; Stone & Vespe, 2022) and its existence in dogs has been shown in experimental models (Allegret et al., 2011). Therefore, the authors chose to treat dogs with ACT_ind_ >1000 s as cases of inaccurate dose estimation, despite achieving our target value of ≥300 s. Although we actively monitored for clinical signs of rebound during the 24‐h postoperative period, we did not conduct any additional laboratory tests such as a late postoperative ACT measurement. Currently, the optimal timeframe (ranging from 2–6 h postoperatively) and preferred methodologies for detecting heparin rebound in human clinical settings remain indecisive as there is limited consensus within literature, as noted by Stone and Vespe (2022). Furthermore, there seems to be a lack of veterinary studies on this topic. However, research on human patients indicates that anti‐Xa testing is a more reliable approach to diagnose heparin rebound in comparison to ACT and aPTT (Stone & Vespe, 2022). The opposite, insufficient anticoagulation (<300 s) after administration of the calculated required heparin dose, occurred just once in this study based on the HDR test. Such instances are even more clinically relevant, as it directly increases the thrombo‐embolic risk. Still, low ACT values allow for the administration of an additional bolus if it can be accurately estimated.

One of the primary reasons for the inability of the HDR test to reliably predict the actual required dose is that this model is simplified and relies on the slope between the first two ACT measurements (so called heparin sensitivity) (Finley & Greenberg, 2013). As suspected from our in vivo findings and later demonstrated by our in vitro data, the relationship between heparin and ACT in characterised by marked variability in individuals and nonlinearity at higher, yet clinically significant heparin concentrations. Numerous factors have been shown to significantly influence heparin sensitivity in humans (Finley & Greenberg, 2013; Garvin et al., 2010; Raymond et al., 2003). The patient's antithrombin concentration is a widely acknowledged determinant of heparin – ACT response (Levy et al., 2000). Antithrombin mediates the anticoagulatory effect of heparin. Therefore, antithrombin deficiency can decrease heparin sensitivity, which may necessitate pretreatment with antithrombin concentrate or fresh frozen plasma (Finley & Greenberg, 2013; Levy et al., 2000). Nevertheless, all dogs in our study exhibited normal antithrombin activity (Kuzi et al., 2010). Platelet deficiency or deficiencies in available coagulation factors such as factor XII, factor XI, factor VIII, factor V, factor II and kallikrein have also been identified as factors that can prolong ACT (Finley & Greenberg 2013; Garvin et al., 2010; Levy et al., 2000). In our study sample, all dogs had a normal platelet count and normal ACT_0_ measurement. Other possible effects of intravascular procedures, including hypothermia and haemodilution, have been found to lead to false prolongation of ACT in human patients (Shore‐Lesserson, 2008; Pelosi et al., 2013). Although there is a chance that these factors may have contributed to the observed ACT measurements exceeding >1000 s, it is improbable that they had a significant impact since only minor intraoperative hypothermia (33.5–34.3°C) was detected in dogs with excessively high (>1000 s) intraprocedural ACT values. Additionally, a standard amount of fluid (5 mL/kg/h NaCl 0.9%) was administered during anaesthesia to negate the possibility of anaesthesia‐associated relative hypovolaemia. Similarly, in human patients, significant effects on ACT are usually only observed in CPB, which is often associated with severe hypothermia and haemodilution, but not in similar electrophysiological procedures (O'Carroll‐Kuehn & Meeran, 2007; Sticherling et al., 2015).

Though the proposed ACT‐based HDR test failed, our research demonstrates considerable concern about the use of fixed‐dose heparin protocols. The observed fluctuations in heparin sensitivity among dogs signify that it is highly improbable that any fixed heparin dose would be fitting for an appropriate proportion of dogs. For example, some reports in dogs suggest a heparin dose of 100 IU/kg for CPB aiming for an ACT response of >400–480 s (Akiyama et al., 2005; Shimamura et al., 2006; Tanaka et al., 2003). Within our study, an IV dose of 100 IU/kg yielded ACT measurements of <155 s in all dogs. Even a cumulative dose of 350 IU/kg (100 IU/kg followed by 250 IU/kg 10 min later), exceeding the loading dose commonly administered to humans (90–200 IU/kg) (Sticherling et al., 2015), did not increase ACT ≥300 s in three dogs. These results demonstrate that the use of fixed‐dose heparin protocols cannot be considered safe in dogs and that the individual effect of heparin must always be closely monitored.

The results of the in vitro study confirmed a strong correlation between heparin concentration and ACT in blood across the range of heparin concentrations tested. However, as suggested by our in vivo data, this relationship was not linear. In half of the samples, a disproportional rise in ACT measurements occurred at a heparin concentration of 4 IU/mL. This finding is of clinical importance, assuming that a heparin concentration of 4 IU/mL corresponds to an IV heparin equivalent dose of 292 IU/kg in a dog (Levy et al., 2000). In addition, even in the absence of potential confounding factors such as haemodilution and hypothermia (the in vitro blood sample was taken at the start of the procedure), considerable interdog variability was observed at the highest heparin concentration (Figure 1).

Finally, in several individual dogs, a disparity between in vitro and in vivo heparin response was observed. For instance, Dog 4 to Dog 7 displayed an ACT response (>2 IU/mL) that was disproportionately high compared to the other four dogs. Interestingly, this subgroup included both the dog with the highest and the lowest in vivo heparin sensitivity. This suggests that a nonlinear response in vitro may not be a reliable predictor of dogs manifesting ACT responses >1000 s during HDR testing in vivo. The latter could be the result of differences in sample processing between in vivo (direct measurement) and in vitro (1/10 sample dilution) and/or the result of the presence of in vivo influences such as heparin sequestration, plasma protein binding and platelet activation (Garvin et al., 2010).

In humans, the laboratory anti‐Xa test has been suggested as a potential substitute to ACT for heparin monitoring during intravascular procedures (Reyher et al., 2016). One of the advantages of measuring anti‐Xa is that it allows the clinician to obtain a direct estimate of the circulating plasma heparin concentration, as opposed to measuring a secondary haemostatic response that can be influenced by various factors mentioned above (Gehrie & Laposata, 2012; Reyher et al., 2016). Our in vitro results are promising and indicate a strong correlation between plasma heparin concentrations and anti‐Xa in dogs. Unlike ACT, the relationship between anti‐Xa and heparin was linear. In addition, far less variability in anti‐Xa response was present between dogs at high heparin concentrations (Figure 2). Unfortunately, there is no POC test for anti‐Xa measurement and laboratory analysis is not widely available. Furthermore, the cost of conducting a single anti‐Xa analysis is higher compared to most ACT tests (Reyher et al., 2016; Thompson et al., 2019). These issues need to be addressed before anti‐Xa can be effectively used to monitor heparin administration during intravascular procedures in dogs.

Our study has several limitations. First, the sample size was limited and second, all dogs in the current study were healthy beagles, which differs from clinical practice. Both were due to the fact that these data reflect our first experience of heparin monitoring with the HDR test. Because of the small number of dogs included in this study and the existence of high variability in heparin sensitivity among the dogs, further prospective studies in a larger number of dogs are needed to confirm our results. Nevertheless, the authors believe that sufficient data were present to make a meaningful conclusion about the absence of effectiveness of the current HDR test in dogs. Third, in the absence of any literature evidence of a linear relationship between heparin and ACT in dogs to support the use of the HDR test, the authors were cautious in administering the calculated required dose of heparin. Five dogs received the maximum dose of 250 IU/kg, followed by additional boluses (up to 250 IU/kg) as needed. While this conservative approach later proved justified, it limited our in vivo study to a descriptive analysis. Lastly, we consistently used single ACT measurements 10 min after bolus administration. On the one hand, this is very common in clinical practice and the choice for a 10 min waiting period was based on guidelines from human medicine (Sticherling et al., 2015). On the other hand, the complex in vivo pharmacokinetics of IV heparin in humans and dogs are not fully understood and it is uncertain with the current data whether the ACT measurement was performed at the time of maximal ACT response (Finley & Greenberg, 2013).

## 5 CONCLUSION

The ACT‐based HDR test provided poor estimates of the required heparin dose needed to achieve a target ACT of ≥300 s in dogs. This is most likely attributable by a nonlinear heparin–ACT response as could be demonstrated in half of the dogs in vitro. At the same time, the observed variability in heparin sensitivity between dogs does not support the use of fixed‐dose heparin protocols and stresses the need for heparin monitoring. Anti‐Xa showed excellent correlation with heparin in vitro and therefore appears to be promising for guiding intravenous heparin administration during intravascular procedures in dogs. However, the absence of a POC test suitable for use in the operating theatre and the lack of established veterinary in vivo target values pose significant challenges that need to be addressed.

## AUTHOR CONTRIBUTIONS

Everyone listed as an author fulfils all four of the ICMJE guidelines for authorship. A.H. wrote the manuscript with input from all authors. All authors have given their final approval to this manuscript. All authors agree to be responsible for all aspects of the work.

## CONFLICT OF INTEREST STATEMENT

None of the authors has any financial or personal relationships that could inappropriately influence or bias the content of this paper.

## FUNDING

Arnaut Hellemans is a PhD fellow of the Flemish Research Foundation grant number 1S78823N.

## ETHICS STATEMENT

This research was approved by the Local Ethics Committee (Faculty of Veterinary Medicine of Ghent University (EC 2021–031).

### PEER REVIEW

The peer review history for this article is available at https://www.webofscience.com/api/gateway/wos/peer‐review/10.1002/vms3.1326.

## Supporting information

Supporting Information

## Data Availability

The data that support the findings of this study are available from the corresponding author upon reasonable request.
