# Supplementary material for: Evaluation of the use of a heparin dose‐response test in dogs to determine the optimal heparin dose during intravascular procedures and assessment of the in vitro heparin response in healthy dogs
Source: Vet Med Sci. 2023 Nov 21;10(3):e1326. doi: 10.1002/vms3.1326 (PMC10951632; doi:10.1002/vms3.1326)
Supplement: Supplementary file 1 — Supporting Information [file VMS3-10-e1326-s001.docx]

| Dog | RBC count  (x10^12/L) | HCT  (%) | PLT count (K/µL) | TP  (g/L) | ALB  (g/L) |
| --- | --- | --- | --- | --- | --- |
| 2 | 8.50 | 55.3 | 311 | 64 | 30 |
| 3 | 7.64 | 42.9 | 256 | 63 | 31 |
| 4 | 7.54 | 49.0 | 171 | 66 | 35 |
| 5 | 8.37 | 54.1 | 214 | 61 | 29 |
| 6 | 7.19 | 45.3 | 301 | 67 | 30 |
| 7 | 9.11 | 58.8 | 179 | 63 | 34 |
| 8 | 7.25 | 47.9 | 494 | 66 | 29 |

**Appendix I.** Summary of several key haematological and biochemical parameters for each dog.

Abbreviations: ALB: albumin; HCT, haematocrit; PLT, platelet; RBC: red blood cell; TP: total protein. Reference interval RBC count: 5.65-8.87 x10^12/L; reference interval HCT = 37.3-61.7%; reference interval PLT: 148-484, reference interval TP: 54-76 g/L; reference interval ALB: 28-43 g/L.
